# Dimethyl [hydr­oxy(2-nitro­phen­yl)meth­yl]phospho­nate

**DOI:** 10.1107/S1600536809005467

**Published:** 2009-02-21

**Authors:** M. Nawaz Tahir, Nurcan Acar, Hamza Yilmaz, Muhammad Ilyas Tariq, Dinçer Ülkü

**Affiliations:** a Department of Physics, University of Sargodha, Sargodha, Pakistan; b Department of Chemistry, Faculty of Science, University of Ankara, Ankara, Turkey; c Department of Chemistry, University of Sargodha, Sargodha, Pakistan; d Department of Physics Engineering, Hacettepe University, Beytepe 06532, Ankara, Turkey

## Abstract

In the title compound, C_9_H_12_NO_6_P, intra­molecular C—H⋯O hydrogen bonds form five- and six-membered rings. In the crystal, inversion dimers lined by pairs of C—H⋯O hydrogen bonds occur with ring motifs *R*_2_^2^(10). The O atom of the hydr­oxy group behaves as an accepter and the benzene ring as donor. Adjacent dimers are connected through O—H⋯O links.

## Related literature

For related structures, see: Acar *et al.* (2009 ▶); Tahir *et al.* (2007 ▶); Chen *et al.* (2008 ▶); Maliha *et al.* (2009 ▶). For hydrogen-bond motifs, see: Bernstein *et al.* (1995 ▶).
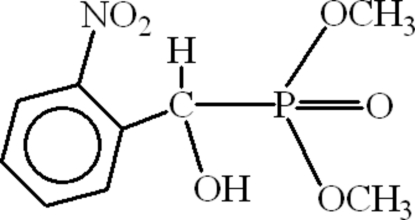


## Experimental

### 

#### Crystal data

C_9_H_12_NO_6_P
*M* *_r_* = 261.17
Monoclinic, 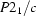

*a* = 9.8685 (12) Å
*b* = 7.5081 (11) Å
*c* = 16.1052 (12) Å
β = 90.341 (1)°
*V* = 1193.3 (2) Å^3^
*Z* = 4
Mo *K*α radiation
μ = 0.25 mm^−1^
*T* = 296 K
0.26 × 0.20 × 0.18 mm

#### Data collection

Enraf–Nonius CAD-4 diffractometer
Absorption correction: ψ scan (*MolEN*; Fair, 1990 ▶) *T*_min_ = 0.939, *T*_max_ = 0.959
2222 measured reflections
2093 independent reflections
1873 reflections with *I* > 2σ(*I*)
*R*_int_ = 0.017
3 standard reflections frequency: 120 min intensity decay: −1.6%

#### Refinement

*R*[*F* ^2^ > 2σ(*F* ^2^)] = 0.057
*wR*(*F* ^2^) = 0.231
*S* = 1.00
2093 reflections
162 parameters
H atoms treated by a mixture of independent and constrained refinement
Δρ_max_ = 0.70 e Å^−3^
Δρ_min_ = −0.50 e Å^−3^

### 

Data collection: *CAD-4 EXPRESS* (Enraf–Nonius, 1992 ▶); cell refinement: *CAD-4 EXPRESS*; data reduction: *MolEN* (Fair, 1990 ▶); program(s) used to solve structure: *SHELXS86* (Sheldrick, 2008 ▶); program(s) used to refine structure: *SHELXL97* (Sheldrick, 2008 ▶); molecular graphics: *ORTEP-3 for Windows* (Farrugia, 1997 ▶) and *PLATON* (Spek, 2009 ▶); software used to prepare material for publication: *WinGX* (Farrugia, 1999 ▶).

## Supplementary Material

Crystal structure: contains datablocks global, I. DOI: 10.1107/S1600536809005467/at2724sup1.cif

Structure factors: contains datablocks I. DOI: 10.1107/S1600536809005467/at2724Isup2.hkl

Additional supplementary materials:  crystallographic information; 3D view; checkCIF report

## Figures and Tables

**Table 1:** Hydrogen-bond geometry (Å, °)

*D*—H⋯*A*	*D*—H	H⋯*A*	*D*⋯*A*	*D*—H⋯*A*
O1—H1⋯O4^i^	0.87 (3)	1.81 (3)	2.674 (3)	171 (4)
C6—H6⋯O1	0.93	2.30	2.688 (3)	104
C6—H6⋯O1^ii^	0.93	2.58	3.343 (4)	140
C7—H7⋯O2	0.93 (3)	2.29 (3)	2.827 (4)	116 (2)

Symmetry codes: (i) 
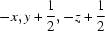
; (ii) 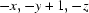.

**Fig. 1.**
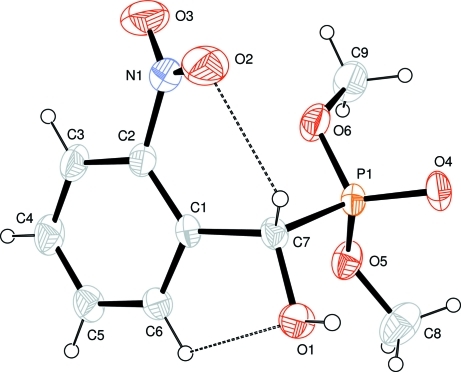
ORTEP drawing of the title compound, (C9H12NO6P), with the atom numbering scheme. The thermal ellpsoids are drawn at the 50% probability level. H-atoms are shown by small circles of arbitrary radii. The broken lines indicate the intermolecular H-bondings.

**Fig. 2.**
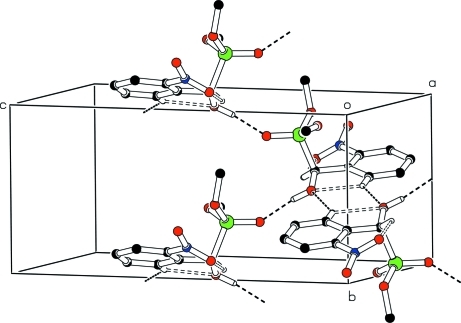
The partial packing figure (PLATON: Spek, 2009) shows the formation of ring motifs through hydrogen bonding.

## supplementary crystallographic information

### Comment

(*R*)-Dimethyl [(2-chlorophenyl)hydroxymethyl]phosphonate (Tahir *et al.*, 2007) and Dimethyl (1-hydroxy-1,2-diphenylethyl)phosphonate (Acar *et al.*, 2009) have been reported by us. In continuation to the study of phosphonate compounds, we herein report the preparation and crystal structure of the title compound (I), (Fig 1).

Diethyl [hydroxy(2-nitrophenyl)methyl]phosphonate (II) (Chen *et al.*, 2008) have also been published which have similar coordination around the C-atom having α-hydroxy group. But it is observed that the change of diethylphosphonate (II) with dimethylphosphonate (I) results in the S-conformation at the methine. In (I), the P═O is 1.467 (2) Å, whereas P–O and P–C have values of [1.557 (2), 1.563 (2) Å] and 1.829 (2) Å, respectively. The nitro group is oriented at an angle of 27.96 (23)° with the benzene ring A(C1—C6). There exist two intramolecular H-bondings which form five B(O1/C7/C1/C6/H6···O1) and six C(O2/N1/C2/C1/C7/H7···O2) membered rings. The title compound is dimerized (Fig 2) forming ring motifs *R*_2_^2^(10) (Bernstein *et al.*, 1995) if only intermolecular H-bonding is concerned. This ten membered ring is splitted into three rings through intramolecular H-bonding resulting in the formation of central four membered ring [O···H···O···H···O]. A similar ring has already been observed in 3-[(methylcarbamoyl)amino]-1*H*-isoindolium chloride (Maliha *et al.*, 2009). The O-atom of hydroxy group behaves as an accepter and the benzene ring as donar. The adjacent dimers are connected through intermolecular H-bonds of O–H···O type, where the accepter is doubly bonded O the phosphonate group.

### Experimental

A solution of *O*-nitrobenzaldehide (3.01 g, 20 mmole) and dimethylphosphonate (2.20 g, 20 mmole) was prepared in THF (50 ml). To this solution, a powder mixture of an equal amount of KF and commercial Al_2_O_3_ (2.5 g + 2.5 g) was added slowly and stirred for 48 h at 273 K. The product was filtered and the filtrate was evaporated at room temperature. The crystalline material obtained after two days was washed with ether and recrystallized in a solution mixture of petroleum ether and THF(1:1), [m.p: 383 K].

### Refinement

All H-atoms appeared in Difference Fourier Map. The coordinations of the atom H7 bounded to the atom C7 and the atom H1 of the hydroxyl group were refined isotropically.

Thermal parameter of these H atoms was taken 1.2 and 1.5 times of the corresponding atoms, respectively.

The H atom (H7) bound to the atom C7 and the H atom (H1) of the hydroxyl group were located in a diffrerence map and their positions refined, with C—H = 0.93 (3) Å and 0.87 (3) Å. The other H atoms were positioned with idealized geometry and refined using a riding model, with C—H distances 0.93–0.96 Å. All H atoms were refined with isotropic displacement parameters (set to 1.2 times of the *U*_eq_ of the parent atom). For methyl and hydroxyl group *U*_iso_(H) = 1.5 *U*_eq_.

### Crystal data

**Table:**

C_9_H_12_NO_6_P	*F*(000) = 544
*M**_r_* = 261.17	*D*_x_ = 1.454 Mg m^−^^3^
Monoclinic, *P*2_1_/*c*	Melting point: 383 K
Hall symbol: -P 2ybc	Mo *K*α radiation, λ = 0.71073 Å
*a* = 9.8685 (12) Å	Cell parameters from 25 reflections
*b* = 7.5081 (11) Å	θ = 11.7–21.0°
*c* = 16.1052 (12) Å	µ = 0.25 mm^−^^1^
β = 90.341 (1)°	*T* = 296 K
*V* = 1193.3 (2) Å^3^	Prismatic, brown
*Z* = 4	0.26 × 0.20 × 0.18 mm

### Data collection

**Table:**

Enraf–Nonius CAD-4 diffractometer	*R*_int_ = 0.017
ω/2θ scans	θ_max_ = 25.0°, θ_min_ = 2.5°
Absorption correction: ψ scan (*MolEN*; Fair, 1990)	*h* = −11→0
*T*_min_ = 0.939, *T*_max_ = 0.959	*k* = −8→0
2222 measured reflections	*l* = −19→19
2093 independent reflections	3 standard reflections every 120 min
1873 reflections with *I* > 2σ(*I*)	intensity decay: −1.6%

### Refinement

**Table:**

Refinement on *F*^2^	Primary atom site location: structure-invariant direct methods
Least-squares matrix: full	Secondary atom site location: difference Fourier map
*R*[*F*^2^ > 2σ(*F*^2^)] = 0.057	H atoms treated by a mixture of independent and constrained refinement
*wR*(*F*^2^) = 0.231	*w* = 1/[σ^2^(*F*_o_^2^) + (0.199*P*)^2^ + 0.3221*P*] where *P* = (*F*_o_^2^ + 2*F*_c_^2^)/3
*S* = 1.00	(Δ/σ)_max_ < 0.001
2093 reflections	Δρ_max_ = 0.70 e Å^−^^3^
162 parameters	Δρ_min_ = −0.50 e Å^−^^3^
0 restraints	

### Special details

**Table:**

Experimental. the structure was solved by Patterson method using *SHELX86* (Sheldrick, 2008); the whole molecule was recognized
Geometry. Bond distances, angles *etc*. have been calculated using the rounded fractional coordinates. All su's are estimated from the variances of the (full) variance-covariance matrix. The cell e.s.d.'s are taken into account in the estimation of distances, angles and torsion angles

### Fractional atomic coordinates and isotropic or equivalent isotropic displacement parameters (Å^2^)

**Table:**

	*x*	*y*	*z*	*U*_iso_*/*U*_eq_	
P1	0.11199 (7)	0.14188 (9)	0.16915 (4)	0.0429 (3)	
O1	0.0588 (2)	0.4641 (3)	0.12234 (12)	0.0541 (7)	
O2	0.4424 (3)	0.3284 (5)	0.20368 (16)	0.0854 (12)	
O3	0.5644 (3)	0.1418 (5)	0.1375 (2)	0.0989 (14)	
O4	0.0391 (2)	0.1450 (3)	0.24822 (14)	0.0574 (8)	
O5	0.0264 (2)	0.0741 (3)	0.09399 (13)	0.0590 (8)	
O6	0.2419 (2)	0.0229 (3)	0.16653 (17)	0.0679 (9)	
N1	0.4732 (2)	0.2487 (4)	0.14047 (16)	0.0590 (9)	
C1	0.2651 (3)	0.3507 (3)	0.06417 (15)	0.0380 (8)	
C2	0.3983 (3)	0.2905 (4)	0.06340 (16)	0.0449 (8)	
C3	0.4703 (3)	0.2673 (5)	−0.0095 (2)	0.0580 (10)	
C4	0.4101 (4)	0.3092 (5)	−0.08461 (19)	0.0639 (11)	
C5	0.2801 (3)	0.3732 (5)	−0.08579 (19)	0.0590 (11)	
C6	0.2083 (3)	0.3926 (4)	−0.01258 (16)	0.0485 (9)	
C7	0.1759 (3)	0.3618 (3)	0.14019 (16)	0.0394 (8)	
C8	−0.1193 (4)	0.0865 (7)	0.0912 (3)	0.0860 (16)	
C9	0.2422 (4)	−0.1632 (5)	0.1836 (3)	0.0822 (14)	
H1	0.031 (4)	0.514 (5)	0.168 (2)	0.0650*	
H3	0.55850	0.22381	−0.00787	0.0697*	
H4	0.45732	0.29422	−0.13392	0.0768*	
H5	0.23957	0.40382	−0.13609	0.0710*	
H6	0.11979	0.43474	−0.01489	0.0582*	
H7	0.220 (3)	0.409 (4)	0.186 (2)	0.0472*	
H8A	−0.15277	0.02578	0.04276	0.1289*	
H8B	−0.14567	0.20944	0.08889	0.1289*	
H8C	−0.15650	0.03237	0.14001	0.1289*	
H9A	0.33363	−0.20263	0.19280	0.1231*	
H9B	0.20387	−0.22632	0.13727	0.1231*	
H9C	0.18939	−0.18618	0.23233	0.1231*	

### Atomic displacement parameters (Å^2^)

**Table:**

	*U*^11^	*U*^22^	*U*^33^	*U*^12^	*U*^13^	*U*^23^
P1	0.0397 (5)	0.0498 (6)	0.0394 (6)	−0.0010 (3)	0.0118 (3)	0.0050 (2)
O1	0.0612 (13)	0.0632 (12)	0.0381 (11)	0.0208 (10)	0.0100 (9)	−0.0029 (8)
O2	0.0730 (17)	0.135 (3)	0.0482 (14)	0.0101 (15)	−0.0100 (12)	−0.0049 (14)
O3	0.0687 (19)	0.134 (3)	0.094 (2)	0.0365 (17)	−0.0079 (17)	0.0130 (18)
O4	0.0558 (13)	0.0733 (14)	0.0432 (12)	−0.0081 (10)	0.0176 (10)	0.0110 (9)
O5	0.0521 (13)	0.0700 (14)	0.0551 (13)	−0.0075 (10)	0.0116 (9)	−0.0146 (10)
O6	0.0453 (12)	0.0551 (13)	0.1034 (19)	0.0036 (9)	0.0211 (11)	0.0248 (12)
N1	0.0437 (13)	0.0792 (18)	0.0541 (15)	−0.0038 (12)	0.0011 (11)	0.0101 (13)
C1	0.0422 (14)	0.0397 (13)	0.0322 (13)	−0.0045 (9)	0.0082 (10)	0.0001 (8)
C2	0.0403 (13)	0.0502 (14)	0.0443 (14)	−0.0063 (11)	0.0059 (10)	0.0011 (11)
C3	0.0421 (15)	0.073 (2)	0.0590 (17)	−0.0008 (13)	0.0181 (13)	−0.0008 (14)
C4	0.0621 (19)	0.086 (2)	0.0439 (16)	−0.0039 (17)	0.0213 (13)	−0.0057 (15)
C5	0.065 (2)	0.080 (2)	0.0322 (14)	−0.0026 (15)	0.0077 (12)	0.0027 (12)
C6	0.0500 (16)	0.0613 (16)	0.0341 (14)	0.0044 (12)	0.0052 (11)	0.0036 (11)
C7	0.0442 (14)	0.0449 (14)	0.0291 (13)	0.0009 (10)	0.0065 (10)	0.0001 (9)
C8	0.056 (2)	0.114 (3)	0.088 (3)	−0.004 (2)	−0.0030 (18)	−0.033 (2)
C9	0.068 (2)	0.060 (2)	0.119 (3)	0.0109 (16)	0.025 (2)	0.025 (2)

### Geometric parameters (Å, °)

**Table:**

P1—O4	1.467 (2)	C3—C4	1.381 (5)
P1—O5	1.557 (2)	C4—C5	1.370 (5)
P1—O6	1.563 (2)	C5—C6	1.387 (4)
P1—C7	1.829 (2)	C3—H3	0.9300
O1—C7	1.416 (3)	C4—H4	0.9300
O2—N1	1.221 (4)	C5—H5	0.9300
O3—N1	1.207 (4)	C6—H6	0.9300
O5—C8	1.441 (4)	C7—H7	0.93 (3)
O6—C9	1.424 (4)	C8—H8A	0.9600
O1—H1	0.87 (3)	C8—H8B	0.9600
N1—C2	1.475 (4)	C8—H8C	0.9600
C1—C6	1.390 (4)	C9—H9A	0.9600
C1—C7	1.515 (4)	C9—H9B	0.9600
C1—C2	1.390 (4)	C9—H9C	0.9600
C2—C3	1.387 (4)		
			
P1···H1^i^	3.14 (3)	N1···O6	2.876 (3)
O1···O4	3.145 (3)	N1···H7	2.87 (3)
O1···O5	2.981 (3)	C2···O6	3.035 (4)
O1···C8	3.372 (5)	C2···C4^ix^	3.566 (5)
O1···O4^ii^	2.674 (3)	C3···C3^ix^	3.556 (5)
O1···C6^iii^	3.343 (4)	C4···C2^ix^	3.566 (5)
O2···C7	2.827 (4)	C6···O1^iii^	3.343 (4)
O2···O6	3.086 (4)	C7···O2	2.827 (4)
O3···C8^iv^	3.240 (5)	C8···O1	3.372 (5)
O4···O1	3.145 (3)	C8···O3^x^	3.240 (5)
O4···O1^i^	2.674 (3)	C8···O5^v^	3.350 (5)
O4···C9^ii^	3.320 (5)	C9···O4^i^	3.320 (5)
O5···C8^v^	3.350 (5)	H1···P1^ii^	3.14 (3)
O5···O1	2.981 (3)	H1···O4^ii^	1.81 (3)
O6···O2	3.086 (4)	H3···O3	2.4200
O6···C2	3.035 (4)	H4···H9A^xi^	2.3800
O6···N1	2.876 (3)	H4···O2^xii^	2.7800
O1···H6^iii^	2.5800	H5···O4^xii^	2.7300
O1···H6	2.3000	H6···O1	2.3000
O1···H8B	2.8300	H6···O1^iii^	2.5800
O1···H9B^vi^	2.7400	H7···O2	2.29 (3)
O2···H7	2.29 (3)	H7···N1	2.87 (3)
O2···H9A^vii^	2.7700	H8A···O5^v^	2.6500
O2···H4^viii^	2.7800	H8B···O1	2.8300
O3···H3	2.4200	H8C···O3^x^	2.8700
O3···H8C^iv^	2.8700	H8C···O4	2.7300
O4···H5^viii^	2.7300	H9A···O2^xiii^	2.7700
O4···H9C	2.9100	H9A···H4^xi^	2.3800
O4···H9C^ii^	2.6100	H9B···O1^xiv^	2.7400
O4···H8C	2.7300	H9C···O4	2.9100
O4···H1^i^	1.81 (3)	H9C···O4^i^	2.6100
O5···H8A^v^	2.6500		
			
O4—P1—O5	114.43 (12)	P1—C7—O1	105.03 (19)
O4—P1—O6	116.05 (14)	C2—C3—H3	120.00
O4—P1—C7	112.25 (13)	C4—C3—H3	120.00
O5—P1—O6	103.49 (13)	C3—C4—H4	120.00
O5—P1—C7	106.44 (12)	C5—C4—H4	120.00
O6—P1—C7	103.00 (13)	C4—C5—H5	120.00
P1—O5—C8	122.7 (2)	C6—C5—H5	120.00
P1—O6—C9	123.8 (2)	C1—C6—H6	119.00
C7—O1—H1	109 (2)	C5—C6—H6	119.00
O2—N1—O3	123.3 (3)	P1—C7—H7	107.7 (19)
O3—N1—C2	118.6 (3)	O1—C7—H7	109.5 (18)
O2—N1—C2	118.1 (3)	C1—C7—H7	113.1 (19)
C2—C1—C7	125.4 (2)	O5—C8—H8A	109.00
C6—C1—C7	118.2 (3)	O5—C8—H8B	109.00
C2—C1—C6	116.2 (2)	O5—C8—H8C	109.00
N1—C2—C3	115.4 (3)	H8A—C8—H8B	109.00
C1—C2—C3	122.5 (3)	H8A—C8—H8C	109.00
N1—C2—C1	122.1 (2)	H8B—C8—H8C	110.00
C2—C3—C4	119.5 (3)	O6—C9—H9A	109.00
C3—C4—C5	119.3 (3)	O6—C9—H9B	110.00
C4—C5—C6	120.5 (3)	O6—C9—H9C	109.00
C1—C6—C5	121.8 (3)	H9A—C9—H9B	109.00
P1—C7—C1	111.07 (16)	H9A—C9—H9C	109.00
O1—C7—C1	110.1 (2)	H9B—C9—H9C	109.00
			
O4—P1—O5—C8	25.6 (3)	C6—C1—C2—N1	177.3 (3)
O6—P1—O5—C8	152.8 (3)	C6—C1—C2—C3	−2.1 (4)
C7—P1—O5—C8	−99.0 (3)	C7—C1—C2—N1	−7.1 (4)
O4—P1—O6—C9	58.0 (3)	C7—C1—C2—C3	173.6 (3)
O5—P1—O6—C9	−68.2 (3)	C2—C1—C6—C5	0.8 (4)
C7—P1—O6—C9	−179.0 (3)	C7—C1—C6—C5	−175.2 (3)
O4—P1—C7—O1	−68.02 (19)	C2—C1—C7—P1	−76.9 (3)
O4—P1—C7—C1	173.00 (18)	C2—C1—C7—O1	167.2 (2)
O5—P1—C7—O1	57.90 (19)	C6—C1—C7—P1	98.6 (2)
O5—P1—C7—C1	−61.1 (2)	C6—C1—C7—O1	−17.3 (3)
O6—P1—C7—O1	166.43 (17)	N1—C2—C3—C4	−177.7 (3)
O6—P1—C7—C1	47.5 (2)	C1—C2—C3—C4	1.7 (5)
O2—N1—C2—C1	−28.4 (4)	C2—C3—C4—C5	0.1 (5)
O2—N1—C2—C3	151.0 (3)	C3—C4—C5—C6	−1.3 (6)
O3—N1—C2—C1	153.8 (3)	C4—C5—C6—C1	0.9 (5)
O3—N1—C2—C3	−26.8 (4)		

Symmetry codes: (i) −*x*, *y*−1/2, −*z*+1/2; (ii) −*x*, *y*+1/2, −*z*+1/2; (iii) −*x*, −*y*+1, −*z*; (iv) *x*+1, *y*, *z*; (v) −*x*, −*y*, −*z*; (vi) *x*, *y*+1, *z*; (vii) −*x*+1, *y*+1/2, −*z*+1/2; (viii) *x*, −*y*+1/2, *z*+1/2; (ix) −*x*+1, −*y*+1, −*z*; (x) *x*−1, *y*, *z*; (xi) −*x*+1, −*y*, −*z*; (xii) *x*, −*y*+1/2, *z*−1/2; (xiii) −*x*+1, *y*−1/2, −*z*+1/2; (xiv) *x*, *y*−1, *z*.

### Hydrogen-bond geometry (Å, °)

**Table:**

*D*—H···*A*	*D*—H	H···*A*	*D*···*A*	*D*—H···*A*
O1—H1···O4^ii^	0.87 (3)	1.81 (3)	2.674 (3)	171 (4)
C6—H6···O1	0.9300	2.3000	2.688 (3)	104.00
C6—H6···O1^iii^	0.9300	2.5800	3.343 (4)	140.00
C7—H7···O2	0.93 (3)	2.29 (3)	2.827 (4)	116 (2)

Symmetry codes: (ii) −*x*, *y*+1/2, −*z*+1/2; (iii) −*x*, −*y*+1, −*z*.
